# Role of Formate Chemoreceptor in *Pseudomonas syringae* pv. *tabaci* 6605 in Tobacco Infection

**DOI:** 10.1264/jsme2.ME25019

**Published:** 2025-07-23

**Authors:** Phuoc Quy Thang Nguyen, Yuta Watanabe, Hidenori Matsui, Nanami Sakata, Yoshiteru Noutoshi, Kazuhiro Toyoda, Yuki Ichinose

**Affiliations:** 1 The Graduate School of Environmental, Life, Natural Science and Technology, Okayama University, Tsushima-naka 1–1–1, Kita-ku, Okayama 700–8530, Japan

**Keywords:** chemoreceptor, formate, *mcpF*, *Pseudomonas syringae*, virulence

## Abstract

Chemotaxis is essential for infection by plant pathogenic bacteria. The causal agent of tobacco wildfire disease, *Pseudomonas syringae* pv. *tabaci* 6605 (*Pta*6605), is known to cause severe leaf disease and is highly motile. The requirement of chemotaxis for infection has been demonstrated through the inoculation of mutant strains lacking chemotaxis sensory component proteins. *Pta*6605 possesses 54 genes that encode chemoreceptors (known as methyl-accepting chemotaxis proteins, MCPs). Chemoreceptors are classified into several groups based on the type and localization of ligand-binding domains (LBD). Cache LBD-type chemoreceptors have been reported to recognize formate in several bacterial species. In the present study, we identified Cache_3 Cache_2 LBD-type Mcp26 encoded by *Pta*6605_RS00335 as a chemoreceptor for formate using a quantitative capillary assay, and named it McpF. Although the deletion mutant of *mcpF* (Δ*mcpF*) retained attraction to 1% yeast extract, its chemotactic response to formate was markedly reduced. Swimming and swarming motilities were also impaired in the mutant. To investigate the effects of McpF on bacterial virulence, we conducted inoculations on tobacco plants using several methods. The Δ*mcpF* mutant exhibited weaker virulence in flood and spray assays than wild-type and complemented strains, highlighting not only the involvement of McpF in formate recognition, but also its critical role in leaf entry during the early stages of infection.

*Pseudomonas syringae* pv. *tabaci* strain 6605 (*Pta*6605) is a Gram-negative hemibiotrophic pathogen that causes wildfire disease in tobacco (Ichinose *et al.*, 2003). The infection cycle of *Pta*6605 begins with an epiphytic phase on the leaf surface. During the early stages of infection, bacteria that have adapted to environmental conditions enter the leaf interior through natural openings, such as stomata or wounds. *Pta*6605 then resides within the leaf apoplast and deploys its virulence factors to evade or suppress the plant’s immune system (Melotto *et al.*, 2006; Xin *et al.*, 2018). The ability of bacteria to enter plant leaves is a critical factor for successful infection. Plant-derived compounds may play a crucial role in facilitating bacterial movement in leaf tissue (Watanabe *et al.*, 2023). Chemotaxis is an ability to move towards an attractant or away from a repellent (Adler, 1966). Therefore, chemotaxis appears to contribute to this stage; bacteria move towards the plant apoplastic fluid where they find and enter stomata. *Pta*6605 is attracted by the apoplastic fluid extracted from both host and non-host plants, indicating that chemotaxis to common plant apoplastic compounds facilitates the entry of bacteria into leaves (Watanabe *et al.*, 2023).

Chemoreceptors known as methyl-accepting chemotaxis proteins (MCPs) play a critical role in the regulation of chemotaxis. A typical chemoreceptor contains a periplasmic ligand-binding domain (LBD), transmembrane domain, histidine kinases, adenylate cyclases, methyl-accepting chemotaxis proteins, and phosphatases (HAMP) domain, and signaling domain. The periplasmic sensing domain may be a LBD, which binds directly with chemoeffectors, or a domain that binds with a chemoeffector-loaded periplasmic protein (Ortega *et al.*, 2017). Fifty-four putative chemoreceptor genes have been identified in *Pta*6605, and their deduced protein structures were classified into four classes based on their amino acid sequence, topology, and localization (Ichinose *et al.*, 2023).

Formic acid (HCOOH), a one-carbon (C1) organic acid, plays an essential role in plants. It may be synthesized through various pathways, including photorespiration, direct CO_2_ reduction, and the decarboxylation of glyoxylate in peroxisomes, chloroplasts, or mitochondria in C3 plants (Hanson and Roje, 2001; Igamberdie and Bykovaa, 1999). Formate appears to contribute to plant metabolism by incorporating TCA cycle-associated organic acids (Zbinovsky and Burris, 1952; Tolbert, 1955; Prabhu and King, 1996). The rapid conversion of formic acid to malic acid and other organic acids has been reported in tobacco leaves, indicating that this is the normal metabolic process for formate in tobacco leaves (Zbinovsky and Burris, 1952).

MCPs for formate have been identified in several animal- and plant-associated bacteria. Atu0526, a single Calcium channels and chemotaxis receptors_3_2 (sCache_3_2) LBD-type MCP in the soil-borne bacterium *Agrobacterium fabrum* C58, binds specifically to formate, but not to acetate, propionate, butyrate, citrate, malate, succinate, fumarate, or salicylate (Wang *et al.*, 2021). PacF, a Cache_3 Cache_2 LBD-type MCP in the phytopathogenic bacterium *Pectobacterium atrosepticum* SCRI1043, also binds to formate at a distal module (Monteagudo-Cascales *et al.*, 2025). A sCache_2 LBD containing the chemoreceptor McpV in *Sinorhizobium meliloti* binds to four carboxylates: acetate, propionate, butyrate, and formate; however, among these carboxylates, formate exhibited the lowest affinity to McpV (Compton *et al.*, 2018). A dCache_1 LBD-type chemoreceptor to formate, Tlp1, was also found in the pathogenic bacterium *Campylobacter jejuni* in poultry. Tlp1 is critical for formate sensing because deletion mutants lost the full chemotactic response to formate (Duan *et al.*, 2023). Despite these findings, the relationship between formate chemotaxis and bacterial virulence has not yet been elucidated. Therefore, we herein aimed to provide insights into the identification of formate chemoreceptors and how they contribute to the motility and virulence of *Pta*6605.

## Materials and Methods

### Bacterial strains and growth conditions

The bacterial strains used in the present study are listed in Table 1. *Escherichia coli* DH5α and S17-1 strains were grown in Luria-Bertani (LB) medium with the appropriate antibiotics at 37°C. *Pta*6605 was cultured in King’s B (KB) medium supplemented with 50‍ ‍μg mL^–1^ nalidixic acid (Nal) at 27°C and minimal medium (MM; 50‍ ‍mM K_2_SO_4_, 7.6‍ ‍mM [NH_4_]_2_SO_4_, 1.7‍ ‍mM MgCl_2_, and 1.7‍ ‍mM NaCl) (Huynh *et al.*, 1989) supplemented with 10‍ ‍mM mannitol and fructose (MMMF). To measure bacterial growth overnight, cultured *Pta*6605 in KB medium was washed, resuspended in fresh medium, and growth was monitored as previously described (Tumewu *et al.*, 2020).

### Generation of mutant and complemented strains

To generate the Δ*mcp26* strain, the *mcp26* gene (*Pta*6605_RS00335) was amplified using the primer pairs listed in Table S1 and inserted into a pUC118-*Hin*cII/BAP vector (Mighty Cloning Reagent Set; Takara Bio). Inverse PCR to delete the open reading frame (ORF) of *mcp26* was followed by digestion with *Bam*HI and then self-ligation and transformation into *E. coli* DH5α cells. The target fragment was subcloned into the mobilizable cloning vector pK18*mobsacB* via the *Eco*RI and *Sph*I sites (Schäfer *et al.*, 1994). The deletion mutant was generated by double homologous recombination as previously described (Tumewu *et al.*, 2022). Recombinant pK18*mobsacB* was introduced into *E. coli S*17-1 and subsequently used for conjugation with the *Pta*6605 wild type (WT). Colonies were selected on KB medium supplemented with 10% sucrose to isolate deletion mutants by second homologous recombination. Δ*mcp26* deletion mutant strains were confirmed by PCR and sequencing. The complemented strain was generated by introducing recombinant pDSK519 (Keen *et al.*, 1988) with the ORF of *mcp26*. Similarly, *mcp24* (RS19225) and *mcp34* (RS11260) were amplified and inserted into a pGEM-T Easy vector (Promega). Inverse PCR to delete the ORFs of *mcp24* and *mcp34* was conducted similarly to *mcp26*, and Δ*mcp24* and Δ*mcp34* were generated. Mutant strains of Δ*mcpG*, Δ*pscA*, Δ*pscB*, Δ*pscC1*, and Δ*pscC2* were previously described (Tumewu *et al.*, 2020, 2021a).

### Quantitative capillary assay

The quantitative capillary method was described in a previous study (Tumewu *et al.*, 2020). Glass capillaries (Drummond Scientific) were flame-sealed at one end and filled with 5‍ ‍μL of either a control or chemoattractant. The negative control was 10‍ ‍mM HEPES (pH 7.4) buffer, and 1% yeast extract (YE) served as the positive control. Formate solution was prepared by diluting formic acid (Wako Chemicals) to the indicated concentration in 10‍ ‍mM HEPES. Capillaries were incubated in 200‍ ‍μL of a bacterial suspension (OD_600_ of 0.05 in 10‍ ‍mM HEPES) at room temperature for 40‍ ‍min. After the incubation, bacteria inside the capillary were suspended in 45‍ ‍μL of 0.9% NaCl, serially diluted, and plated for colony counting.

### Plant growth and virulence assays

Three inoculation methods were used to assess bacterial virulence in tobacco (*Nicotiana tabacum* L. var. Xanthi NC). The flood inoculation method was modified for tobacco plants (Ishiga *et al.*, 2017; Tumewu *et al.*, 2020). Tobacco seedlings were cultivated in Murashige-Skoog (MS) plates containing 0.8% agar, 0.1% sucrose, and vitamins (3‍ ‍mg L^–1^ thiamin hydrochloride, 5‍ ‍mg L^–1^ nicotinic acid, and 0.5‍ ‍mg L^–1^ pyridoxine hydrochloride). Seedlings were inoculated for 10‍ ‍s with 10‍ ‍mL of a bacterial suspension at OD_600_ of 0.004 (8×10^6^ CFU mL^–1^) in 10‍ ‍mM MgSO_4_ and 0.025% Silwet L-77 (OSI Specialties). After decanting the excess inoculum and drying for 30‍ ‍min in a cabinet, plants were incubated at 22°C under 16/8‍ ‍h light/dark conditions. To quantify bacterial growth, two leaves per seedling were sampled; leaf disks were obtained, ground, diluted, and plated on KB-Nal medium. Bacterial colonies were counted to examine bacterial populations in the leaves.

In the spray inoculation method, 8- to 9-week-old plants grown in soil at 25°C under 12/12‍ ‍h light/dark conditions were sprayed with a bacterial suspension (4×10^8^ CFU mL^–1^ in 10‍ ‍mM MgSO_4_ with 0.04% Silwet L-77) and were then incubated under high humidity conditions for 7 days. In the infiltration inoculation method, attached leaves were infiltrated with bacterial suspensions (2×10^5^ CFU mL^–1^ in 10‍ ‍mM MgSO_4_) using a needleless 1-ml syringe. Plants were incubated at 22°C under 16/8‍ ‍h light/dark conditions and symptoms were monitored for 7 days.

### Surface motility assay

Swarming and swimming assays were performed to assess bacterial surface motility (Taguchi and Ichinose, 2011). MMMF medium with 0.25% agar (Bacto agar; Becton, Dickinson and Company) was used for the swimming assay, while 0.4% agar (Bacto agar) SWM medium (0.5% peptone, 0.3% YE) was used for the swarming assay. Bacteria were grown at 27°C overnight in LB containing 10‍ ‍mM MgCl_2_, resuspended in 10‍ ‍mM MgSO_4_, and adjusted to an OD_600_ of 0.3. Two microliters of a bacterial suspension was injected and spotted at the center of MMMF or SWM plates for the swimming and swarming assays, respectively. MMMF plates were incubated at 23°C for 72 h, while SWM plates were incubated at 27°C for 24 h. The spread of the bacterial halo was measured using ImageJ software (Fiji Distribution, version 1.54f).

### Bioinformatics

The protein domain architecture was predicted using the SMART tool (Letunic and Bork, 2018). Multiple alignment was performed using MAFFT software (version 7) (Katoh *et al.*, 2019), and a phylogenetic tree was constructed using Mega software (version 11) (Tamura *et al.*, 2021).

### Statistical ana­lysis

All statistical ana­lyses were performed using GraphPad Prism (version 10.4.1, GraphPad Software). A one-way ana­lysis of variance (ANOVA) was followed by Dunnet’s post hoc test. The confidence level was set at 95%, and *P*<0.05 indicated a significant difference.

## Results

### Chemotaxis of Pta6605 to formate

**Fig. 1.**
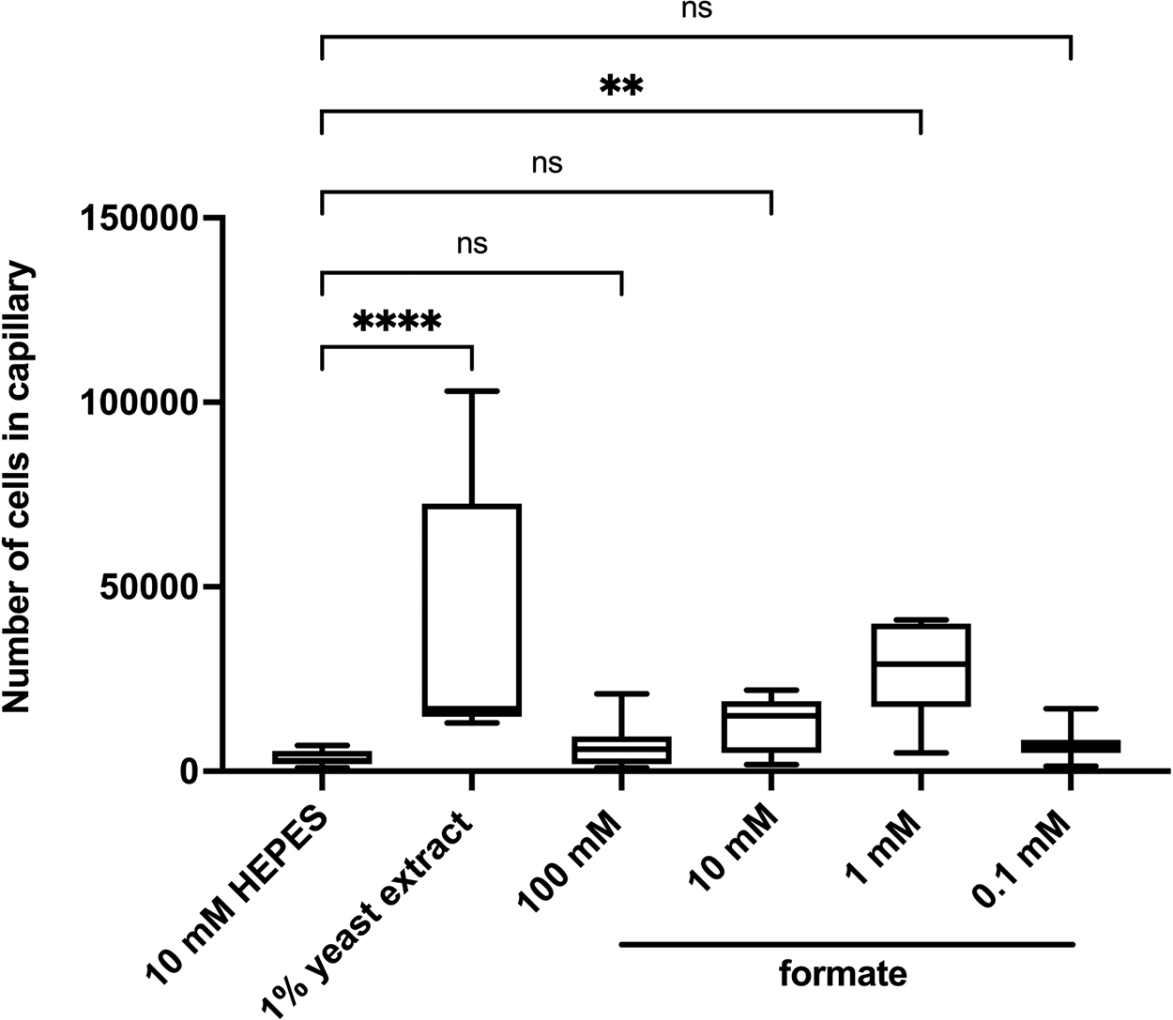
Dose-dependent chemotactic response to formate in *Pta*6605 Chemotaxis to formate was measured by a quantitative capillary assay. As negative and positive controls, 10‍ ‍mM HEPES buffer and 1% yeast extract (YE), respectively, were used. Data represent the number of bacterial cells in each capillary from three independent experiments in triplicate. Asterisks indicate significant differences from the negative control by a one-way ANOVA followed by Dunnett’s multiple comparison test (****P<0.0001, ***P<0.001, **P<0.01, *P<0.05, and ns=not significantly different).

To investigate whether formate is a chemoattractant of *Pta*6605, a quantitative capillary assay was performed with a range of formate concentrations (100, 10, 1, and 0.1‍ ‍mM). To evaluate chemoattraction, the number of bacteria in the capillaries containing the attractant was compared with that in capillaries containing 10‍ ‍mM HEPES buffer. Among the different concentrations tested, *Pta*6605 exhibited a significant chemotactic response to 1‍ ‍mM formate (Fig. 1).

### Comparison of LBDs of formate chemoreceptors and Cache domain-containing MCPs in Pta6605

**Fig. 2.**
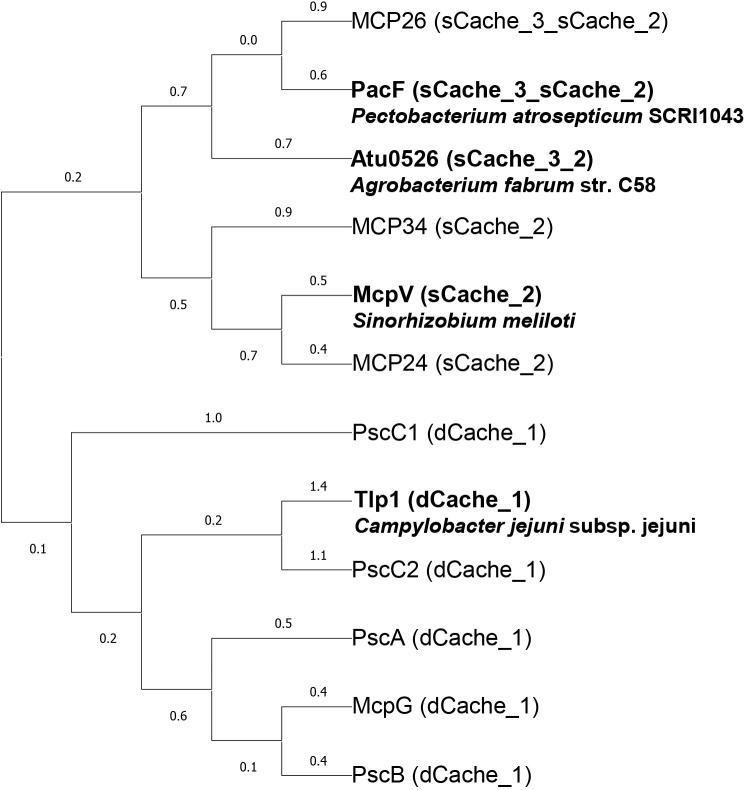
Maximum likelihood tree based on the ligand-binding domain (LBD) of previously known formate chemoreceptors and their homologs in *Pta*6605 Multiple alignment was performed using MAFFT software (version 7) (Katoh *et al.*, 2019). A phylogenetic tree was created using Mega software (version 11) (Tamura *et al.*, 2021).

MCPs with various types of LBDs have been identified as bacterial formate chemoreceptors. Several formate chemoreceptors have been reported to date. Based on previous findings, MCPs with Cache domain-containing LBD appear to be formate chemoreceptors. Since eight Cache domain-containing MCPs are present in *Pta*6605 (Ichinose *et al.*, 2023), they were selected as candidates for formate chemoreceptors and included Cache_3 Cache_2 domain-containing Mcp26 (encoded in RS00335 and tentatively numbered), sCache_2 domain-containing Mcp24 (encoded in RS19225) and Mcp34 (encoded in RS11260), and dCache domain-containing McpG, PscA, PscB, PscC1, and PscC2 (Tumewu *et al.*, 2020, 2021a). The amino acid sequences of the LBD described above were compared using a maximum likelihood tree (Fig. 2). Based on the results obtained, all of the candidates were considered to be possible formate chemoreceptors.

### Mcp26 is a specific chemoreceptor for formate in Pta6605

**Fig. 3.**
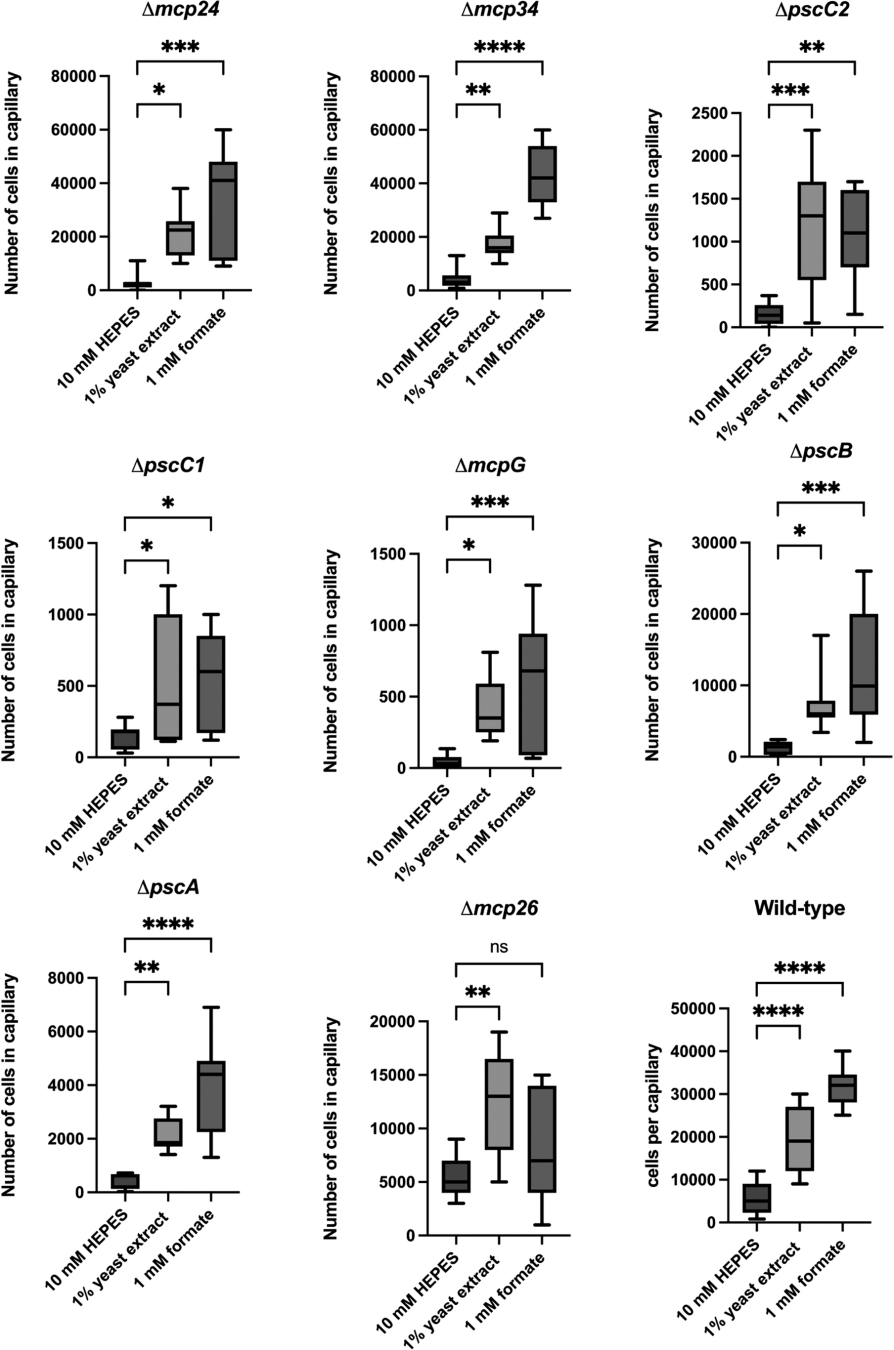
Chemotactic response to formate in deletion mutants for the Cache LBD-containing *mcp* gene in *Pta*6605 Chemotaxis to formate was measured by a quantitative capillary assay to 1‍ ‍mM formate. Data represent the number of bacteria in each capillary from three independent experiments in triplicate. Statistical ana­lyses and data presentation are the same as in Fig. 1.

**Fig. 4.**
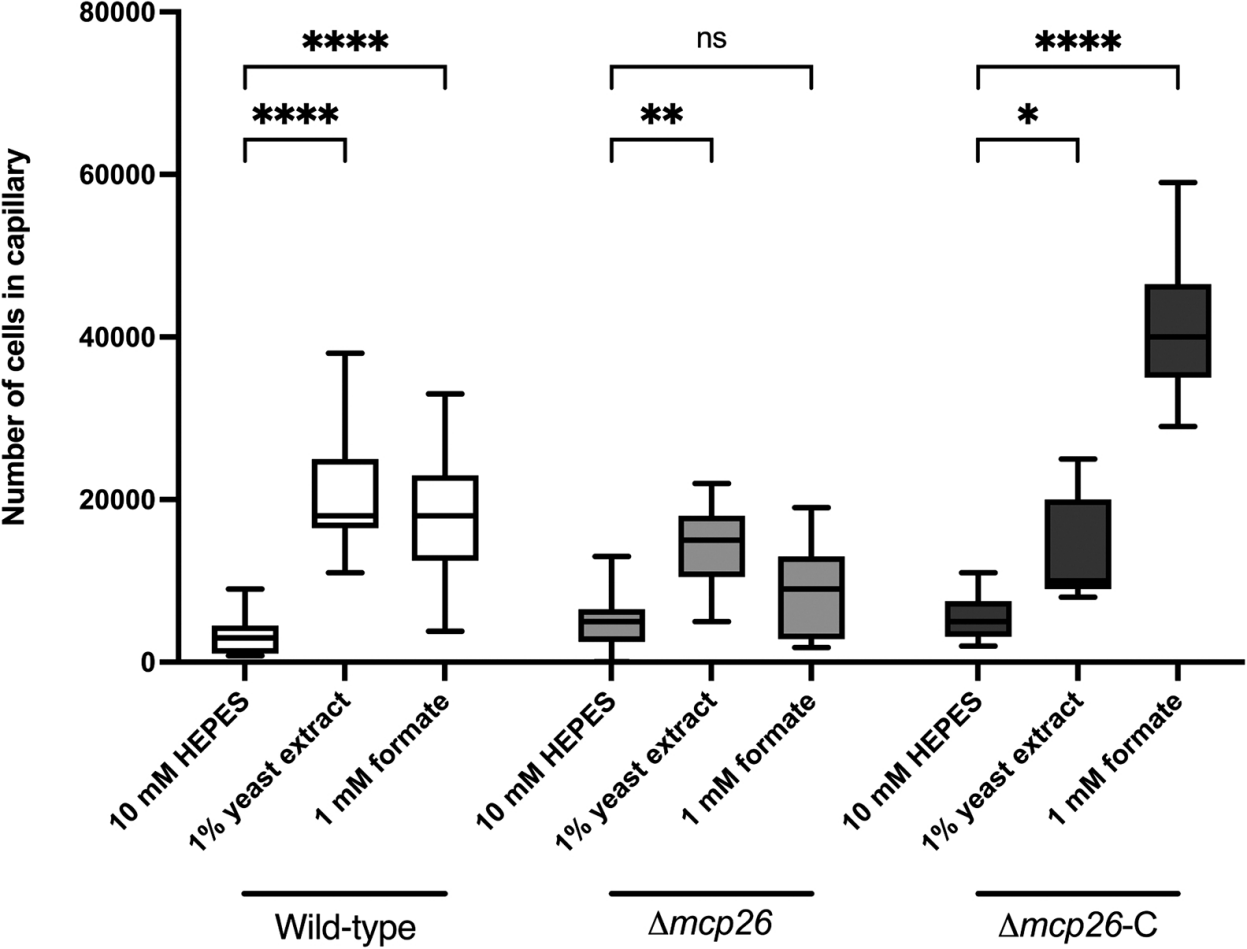
Chemotactic responses to formate of WT, Δ*mcp26*, and Δ*mcp26*-C in *Pta*6605 Chemotaxis to formate was measured by a quantitative capillary assay to 1‍ ‍mM formate. Data represent the number of bacteria in each capillary from three independent experiments in triplicate. Statistical ana­lyses and data presentation are the same as in Fig. 1.

The chemotactic response of each deletion mutant to 1‍ ‍mM formate was assessed using quantitative capillary assays. As shown in Fig. 3, all mutant strains, except for the Δ*mcp26* strain, exhibited chemotaxis to formate. The Δ*mcp26* strain did not show a significant attraction to 1‍ ‍mM formate, but was still attracted to 1% YE. Although the chemotactic response of the Δ*mcp26* mutant to formate varied, only Δ*mcp26* exhibited a significant reduction in formate attraction among the mutants tested (Fig. 3). This variability may be attributed to the presence of other environmental factors, such as inorganic ions, a pH gradient (pH taxis), or oxygen levels (aerotaxis) (Matilla and Krell, 2018), which were difficult to fully control under experimental conditions and may have affected formate chemotaxis. To confirm the regulation of Mcp26 in chemotaxis to formate, a complemented strain, Δ*mcp26-*C, carrying *mcp26* on the plasmid was generated. As expected, formate sensing was restored in Δ*mcp26-*C, and the attraction level was even higher than in WT (Fig. 4). Based on these results, we designated Mcp26 as the specific formate chemoreceptor, McpF, in *Pta*6605.

### Surface motility

**Fig. 5.**
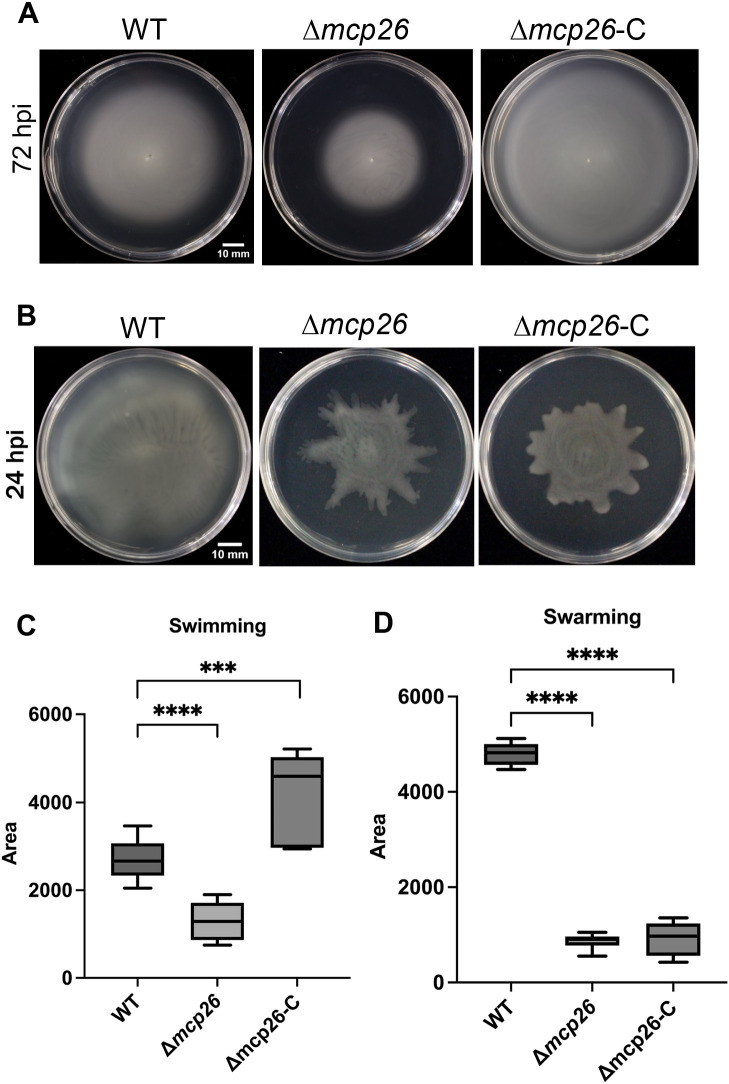
Surface motility tests Bacterial motility was assessed after an incubation for 72‍ ‍h using a swimming assay (A) and 24‍ ‍h using a swarming assay (B). The spread areas of each strain were measured using ImageJ software for the swimming (C) and swarming (D) tests. Statistical ana­lyses and data presentation are the same as in Fig. 1.

The Δ*mcp26* mutant exhibited reduced swimming and swarming motilities (Fig. 5). In the complemented strain, only swimming motility was restored. To investigate the effects of formate on swimming motility, we supplied 1‍ ‍mM formate to MMMF medium. The motilities of both WT and Δ*mcp26* were not affected by formate (Fig. S1). In addition to moving them towards a favorable environment, chemotaxis enables bacteria to detect and access a nutrient pool (Matilla *et al.*, 2023). The expansion of bacterial halos may‍ ‍be affected by their ability to utilize carbon sources. Swimming tests were performed using MM medium with 1‍ ‍mM formate to evaluate the ability of *Pta*6605 to utilize formate as a carbon source. Neither WT nor Δ*mcp26* grew in MM medium without carbon sources other than formate (Fig. S2), indicating that *Pta*6605 cannot use formate as a unique carbon source for metabolism.

### Virulence of mutants on host tobacco leaves

**Fig. 6.**
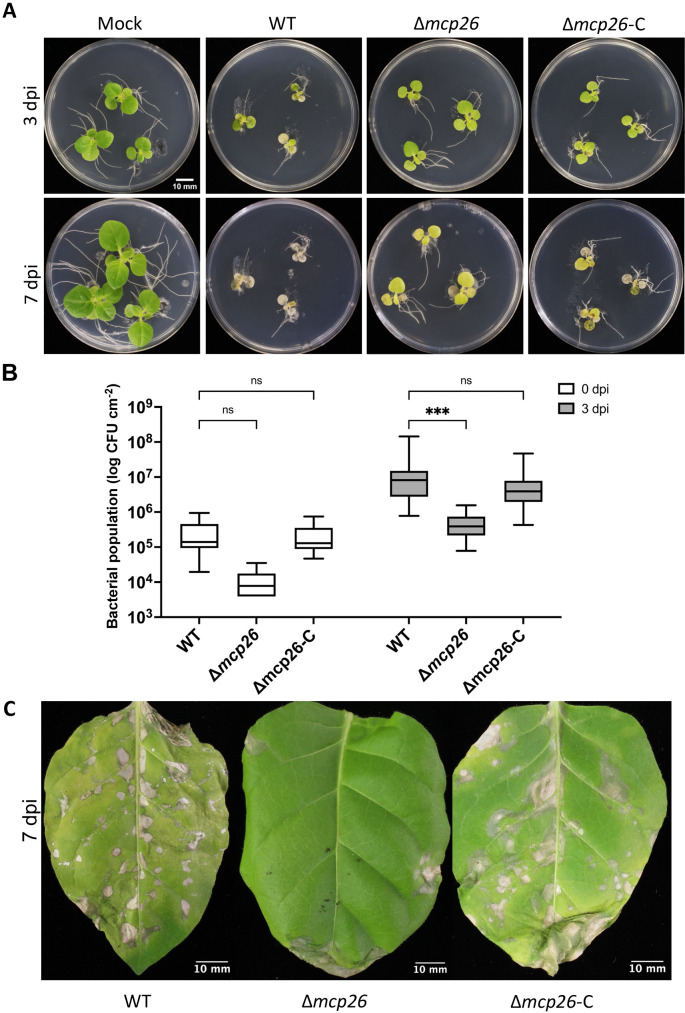
Inoculation of tobacco host plants (A) Tobacco seedlings were flood-inoculated with WT, Δ*mcp26*, or Δ*mcp26*-C. Representative photos were taken at 3 and 7 dpi. (B) Bacterial populations in leaves were quantified at 3 hpi (0 dpi) and 3 dpi. Statistical ana­lyses and data presentation are the same as in Fig. 1. The inoculation was performed with three independent repeats (*n*=3 for 0 dpi, *n*=8 for 3 dpi). (C) Tobacco leaves were sprayed with a bacterial suspension at a density 4×10^8^ CFU mL^–1^ in 10‍ ‍mM MgSO_4_ and 0.04% Silwet L-77. Symptoms were observed at 7 dpi. The bar is 10‍ ‍mm.

The flood inoculation method was used to assess bacterial virulence by evaluating disease symptoms and bacterial populations inside the leaves (Fig. 6A and B). The population of the Δ*mcp26* mutant was significantly smaller than that of the WT strain at three days post inoculation (dpi), whereas the number of complemented strains recovered to WT levels (Fig. 6B). Since motility affects successful infection by pathogenic bacteria, reduced motility may hinder entry into the leaf apoplast, thereby markedly affecting bacterial multiplication. The deletion of *mcp26* (*mcpF*) resulted in reductions in swimming and swarming motilities, suggesting the impaired leaf invasion ability of the mutant. Furthermore, no significant differences in growth were observed among three strains in the culture (Fig. S3), indicating that differences in the bacterial population inside the leaves were not driven by bacterial growth. Consistent with bacterial growth, disease symptoms in tobacco leaves were severe when inoculated with the WT and Δ*mcp26*-C strains (Fig. 6A). The symptoms of the leaves inoculated with Δ*mcp26* were also severe; however, the onset of symptoms was slower than with the inoculation with the WT strain. In addition, disease symptoms following the spray inoculation were consistent with those observed after the flood inoculation, where leaves inoculated with the WT and complemented strains exhibited more severe symptoms than those inoculated with the mutant (Fig. 6C). These outcomes suggest that McpF (Mcp26) deficiency reduced bacterial virulence by limiting leaf entry, but not invasiveness. To further investigate this hypothesis, we conducted an infiltration inoculation by directly injecting a bacterial suspension into the leaves. Disease symptoms were observed in leaves infected with all strains at 7 dpi, indicating that the mutant remained virulent and capable of causing disease in the tobacco host plant (Fig. S4).

## Discussion

The Cache domain is the predominant extracellular LBD of chemoreceptors in prokaryotes (Upadhyay *et al.*, 2016). Its structure is characterized by a Per/Arnt/Sim (PAS)-like module and long N-terminal α-helix (Ortega *et al.*, 2017). McpF (Mcp26) was identified as a unique Cache_3 Cache_2 LBD-type chemoreceptor that responds to formate, while sCache_2 and dCache_1 LDB were not involved in this recognition in *Pta*6605. The amino acid sequence of LBD of McpF is homologous to that of PacF in *P. atrosepticum* SCRI1043 and Atu0526 in *A. fabrum* C58 (Fig. S5). A conserved arginine residue (R115) in Atu0526 was previously shown to directly bind to formate and function in the regulation of chemotaxis (Wang *et al.*, 2021). Modifications to a conserved arginine residue (R142) and threonine residues (T145 and T158) in PacF was found to affect formate binding (Monteagudo-Cascales *et al.*, 2025). A multi-alignment ana­lysis of McpF with Atu0526 and PacF revealed a conserved R139 in McpF; therefore, further studies on R139 binding with formate at the membrane distal module and its response to formate are warranted (Fig. S5).

Research on the role of formate in the plant apoplast is limited. Formate was previously detected at low concentrations in the apoplastic fluid of leaves from some Fragaceae plants (Gabriel and Kesselmeier, 1999). Small formate pools in plants were typically in the range of 0.1 to 1‍ ‍μmol g^–1^ fresh weight (Hanson and Roje, 2001). The presence of formate may be associated with its incorporation into other‍ ‍organic acids involved in plant metabolism (Zbinovsky and Burris, 1952). Plants release formic acid into the atmosphere, with its emission from terrestrial vegetation accounting for 3% of global formic acid emissions (Hanson and Roje, 2001). The directional emission of gaseous formic acid via the stomata has been universally observed in higher plants, and the apoplast is the site at which formate is transformed from a liquid to its gas form (Gabriel *et al.*, 1999; Seco *et al.*, 2007; Mochizuki and Tani, 2021), apart from a few crops including *Zea mays*, *Pisum sativum*, *Hordeum vulgare*, and *Avena sativa* (Kesselmeier *et al.*, 1998). These findings support the hypothesis that the presence of formate in stomata and apoplasts may be related to interactions between plants and phyllosphere bacteria.

The relationship between chemoreceptors and bacterial swimming ability is complex and has not yet been fully elucidated. In the case of *Pta*6605, reduced motility was observed in a mutant lacking amino acid chemoreceptor genes (Tumewu *et al.*, 2021a). Chemotaxis systems are functionally classified into three groups: those involved in controlling flagellar motility (F), which includes 17 distinct classes (F1–F17), those regulating type IV pilus’ motility, and those associated with alternative cellular functions unrelated to motility (Wuichet and Zhulin, 2010). In addition, chemosensory signaling pathways typically involve a chemoreceptor and two-component histidine kinase complex, consisting of the kinase CheA and the response regulator CheY, which modulates bacterial flagellar rotation. In *Pta*6605, the deletion of *cheY2* completely abolished swimming and swarming motilities, indicating the crucial role of CheY2 in chemotaxis and flagellar-mediated motility (Tumewu *et al.*, 2021b). Since CheY2 in *Pta*6605 has been linked to the F6 chemosensory pathway (Ichinose *et al.*, 2023), we hypothesized that Mcp26 may be associated with a flagellar-dependent chemotaxis pathway, contributing to the regulation of bacterial motility. Interestingly, while CheY2 is critical for swimming and swarming, we found that *cheY2* expression levels were similar in the Δ*mcp26* mutant and WT strain (data not shown). Therefore, motility regulated by Mcp26 may not be solely dependent on *cheY2* expression and may involve additional signaling mechanisms or unknown chemotaxis pathways. On the other hand, the complemented strain restored abilities for formate sensing and swimming motility, but not swarming motility, suggesting a polar effect caused by the deletion mutation.

The chemotaxis of pathogenic bacteria facilitates initial entry into the plant apoplast during the early stages of infection (Matilla and Krell, 2018). Among 54 MCPs, eight MCPs and their respective ligands have been investigated, with six being shown to contribute to the virulence of *Pta*6605 (Tumewu *et al.*, 2020, 2021a, 2022). McpG, a specific chemoreceptor of γ-aminobutyric acid (GABA), has been reported to affect both the early and late stages of infection (Tumewu *et al.*, 2020). Additionally, among the four other dCache_1 LBD-type MCPs, PscB, PscC1, and PscC2, which are responsible for chemotaxis towards proteinogenic amino acids, excluding tyrosine, are required for the full virulence of *Pta*6605. The deletion of these genes results in the abolishment of virulence due to the complete loss of motility (Tumewu *et al.*, 2021a). Furthermore, AerA and AerB have been shown to meditate aerotaxis, with AerA playing an important role in host infection (Tumewu *et al.*, 2022). Beyond the six MCPs previously associated with virulence, the present study revealed that McpF was also required for leaf entry in both the flood and spray inoculation methods. However, when bacteria were directly introduced into the apoplast via infiltration, the mutant maintained full pathogenicity. This phenotype may be linked to the decreased swimming and swarming motilities of the mutant, which affected its ability to enter host leaves. Moreover, the present results suggest that chemotaxis towards formate plays a crucial role in the early stage of infection in *Pta*6605.

In conclusion, the present study suggests that McpF (Mcp26), which possesses the Cache_3 Cache_2 type LBD, serves as a specific chemoreceptor for formate in *Pta*6605. The absence of McpF reduces virulence in tobacco plant leaves, which is attributed to diminished motility.

## Citation

Nguyen, P. Q. T., Watanabe, Y., Matsui, H., Sakata, N., Noutoshi, Y., Toyoda, K., and Ichinose, Y. (2025) Role of Formate Chemoreceptor in *Pseudomonas syringae* pv. *tabaci* 6605 in Tobacco Infection. *Microbes Environ ***40**: ME25019.

https://doi.org/10.1264/jsme2.ME25019

## Supplementary Material

Supplementary Material

## Figures and Tables

**Table 1.** Bacteria strains and plasmids used in the present study

Bacterial strain, plasmid	Relevant characteristics	Reference or source
*Escherichia coli*		
DH5α	*F–λ–ϕ80dLacZ *Δ*M15 *Δ(*lacZYA-argF*)* U169 recA1 endA1 hsdR17 (rK–mK^+^) supE44 thi-1 gyrA relA1*	Nippon Gene
S17-1	*thi pro hsdR hsdR hsdM^+^ recA *(*chr::RP4-2-Tc::Mu-Km::Tn7*)	Schäfer *et al.*, 1994
*Pseudomonas syringae* pv. *tabaci*		
Isolate 6605	Wild type isolated from tobacco, Nal^r^	Ichinose *et al.*, 2003
Δ*pscA*	Isolate 6605 ΔRS16340, Nal^r^	Tumewu *et al.*, 2021a
Δ*pscB*	Isolate 6605 ΔRS23495, Nal^r^	Tumewu *et al.*, 2021a
Δ*pscC1*	Isolate 6605 ΔRS16480, Nal^r^	Tumewu *et al.*, 2021a
Δ*pscC2*	Isolate 6605 ΔRS11960, Nal^r^	Tumewu *et al.*, 2021a
Δ*mcpG*	Isolate 6605 ΔRS09525, Nal^r^	Tumewu *et al.*, 2020
Δ*mcp24*	Isolate 6605 ΔRS19225, Nal^r^	This study
Δ*mcp34*	Isolate 6605 ΔRS11260, Nal^r^	This study
Δ*mcp26*	Isolate 6605 ΔRS00335, Nal^r^	This study
Δ*mcp26*-C	Isolate 6605 Δ*mcp26*, pD-*mcp26* complemented, Nal^r^, Km^r^	This study
Plasmid		
pUC118	Cloning vector, Amp^r^	Takara Bio
pUC-*mcp26*	*mcp26* fragment containing pUC118, Amp^r^	This study
pGEM-T Easy	Cloning vector, Amp^r^	Promega
pG-*mcp24*	*mcp24* fragment containing pGEM-T Easy, Amp^r^	This study
pG-*mcp34*	*mcp34* fragment containing pGEM-T Easy, Amp^r^	This study
pK18*mobSacB*	Small mobilizable vector, Km^r^, sucrose sensitive (*sacB*)	Schäfer *et al.*, 1994
pK18-Δ*mcp26*	*mcp26* deleted DNA-containing pK18*mob*sacB, Km^r^	This study
pK18-Δ*mcp24*	*mcp24* deleted DNA-containing pK18*mob*sacB, Km^r^	This study
pK18-Δ*mcp34*	*mcp34* deleted DNA-containing pK18*mob*sacB, Km^r^	This study
pDSK519	Broad host range cloning vector	Keen *et al.*, 1988
pD-*mcp26*	pDSK519 possessing expressible *mcp26*, Km^r^	This study

Nal^r^: nalidixic acid resistant; Amp^r^: ampicillin resistant; Km^r^: kanamycin resistant.
