# Supplementary material for: Role of Formate Chemoreceptor in Pseudomonas syringae pv. tabaci 6605 in Tobacco Infection
Source: Microbes Environ. 2025 Jul 23;40(3):ME25019. doi: 10.1264/jsme2.ME25019 (PMC12501870; doi:10.1264/jsme2.ME25019)
Supplement: Supplementary file 1 — Supplementary Material [file 40_25019_s1.pdf]

Supplementary Files

Role of Formate Chemoreceptor in *Pseudomonas syringae* pv. *tabaci* 6605 in Tobacco Infection

Phuoc Quy Thang Nguyen[1], Yuta Watanabe[1], Hidenori Matsui[1], Nanami Sakata[1], Yoshiteru Noutoshi[1], Kazuhiro Toyoda[1], and Yuki Ichinose[1]*

[1]The Graduate School of Environmental, Life, Natural Science and Technology, Okayama University, Tsushima-naka 1–1–1, Kita-ku, Okayama 700–8530, Japan

*Corresponding author: E-mail: yuki@okayama-u.ac.jp

Table S1 Primers used in this study

Primer name	Sequence (5'-3')	Description
mcp26_1	GACTTTACTCTGGAGCCTTTCG	PCR amplification of *mcp26* and surrounding region
mcp26_2	ACGCGGAGCATCGGCACGAT	
mcp26_C1	cgggatccGACTTTACTCTGGAGCCTTTCG	PCR amplification of *mcp26* and surrounding region to generate complement strain
mcp26_C2	cgggatccACGCGGAGCATCGGCACGAT	
mcp26_3	ccgggatccAGAGACTCCATGCCGAATGA	Inverse PCR to delete *mcp26* ORF
mcp26_4	ccgggatccGCCACGTCGATGAACATCG	
mcp24_1	TCATGAGCAGTATCCGCGAG	PCR amplification of *mcp24* and surrounding region
mcp24_2	TCGACACCGGTCAGACCATT	
mcp24_3	ggtctagaACAAGAATCAGCCACAGACG	Inverse PCR to delete *mcp24* ORF
mcp24_4	ggtctagaAGAACTGAACGGGCTGCTGA	
mcp34_1	gggggatccTTGAATCCATACAACGGCGTCTGA	PCR amplification of *mcp34* and surrounding region
mcp34_2	gggggatccTTAGCCTGGCAGCCTGACGG	
mcp34_3	taatctagaTGCTTGCGTGGCAGGGGCGC	Inverse PCR to delete *mcp34* ORF
mcp34_4	gcatctagaTTATGGAGCAGTGCGCTCG	

Lowercase letters indicate artificial nucleotide sequences for *Bam*HI in mcp26_C1, mcp26_C2, mcp26_3, mcp26_4, mcp34_1 and mcp34_2, and for *Xba*I in mcp24_3, mcp24_4, mcp34_3 and mcp34_4.

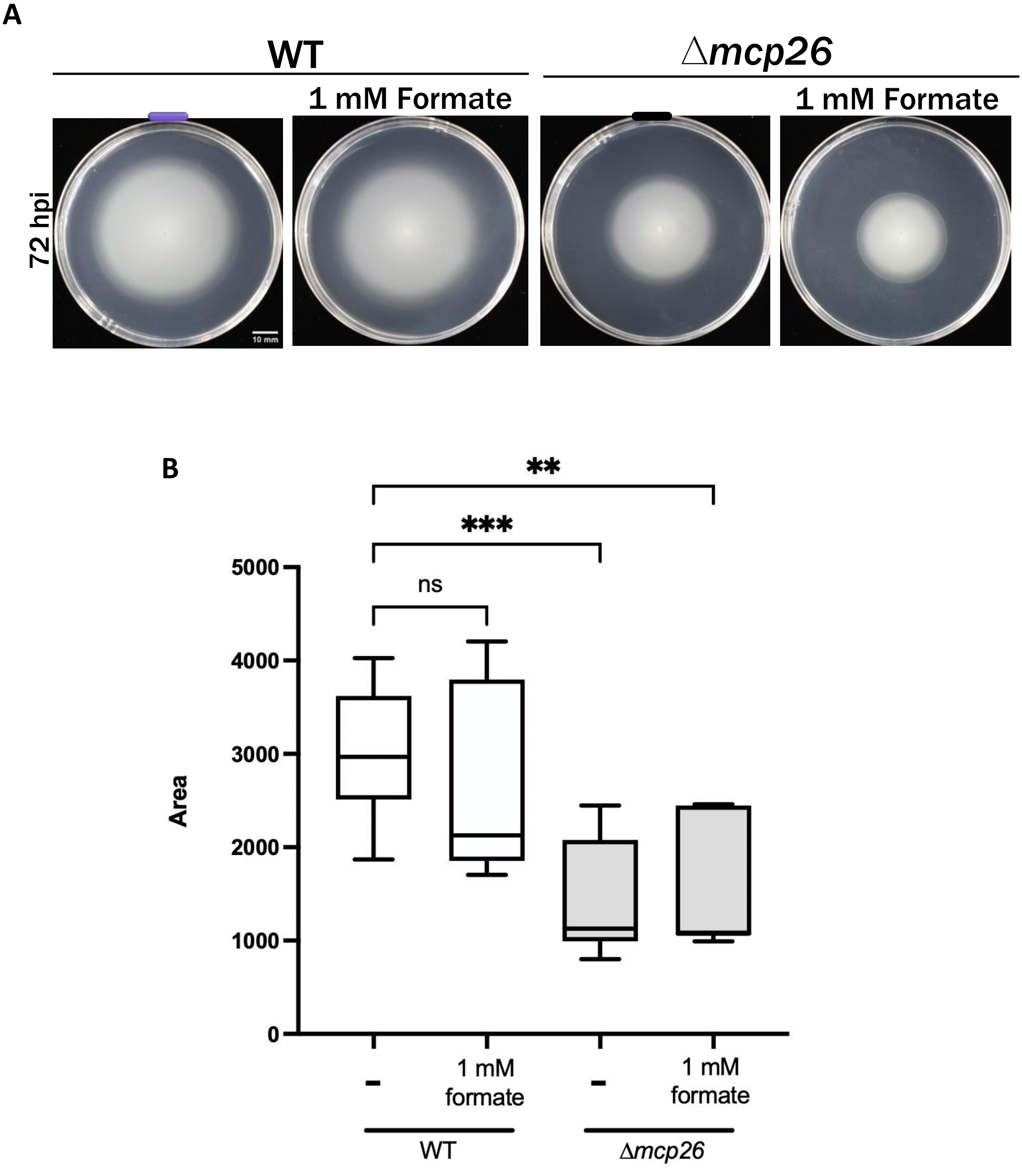

Fig. S1 Effect of formate on swimming motility of WT and Δ*mcp26*

Effect of 1 mM formate was assessed by the addition to MMMF medium (0.25% agar). Bacteria were incubated for 72 h. Bacterial spread areas were measured by imageJ in three different experiments (n=3). Asterisks indicate significant differences from the negative control by one-way ANOVA followed by Dunnett's multiple comparison test (***P<0.001, **P<0.01, and ns = not significantly different).

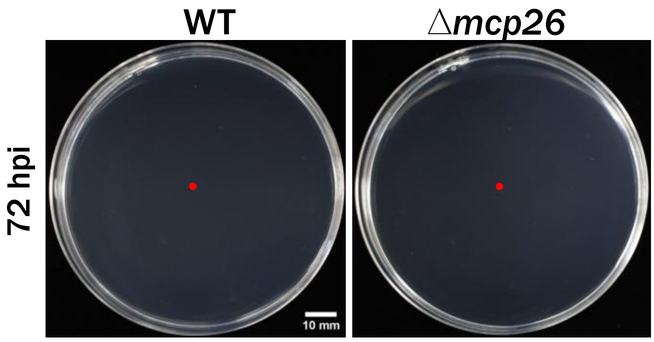

Fig. S2 Swimming motility assay of WT and Δ*mcp26* on MM medium (0.25% agar) supplemented with 1 mM formate

Red dots imply bacteria injection sites.

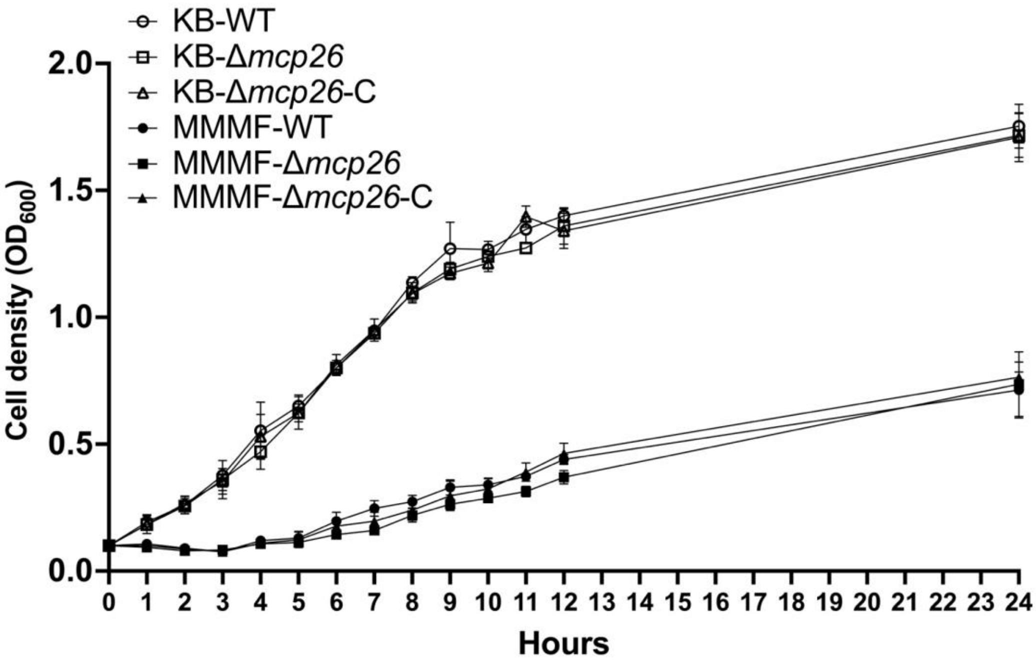

Fig. S3 Bacterial growth in KB and MMMF media

Bacterial growth was monitored among the WT, Δ*mcp26* and Δ*mcp26*-C strains cultured in KB and MMMF media. Error bars represent standard error from two independent experiments in triplicate.

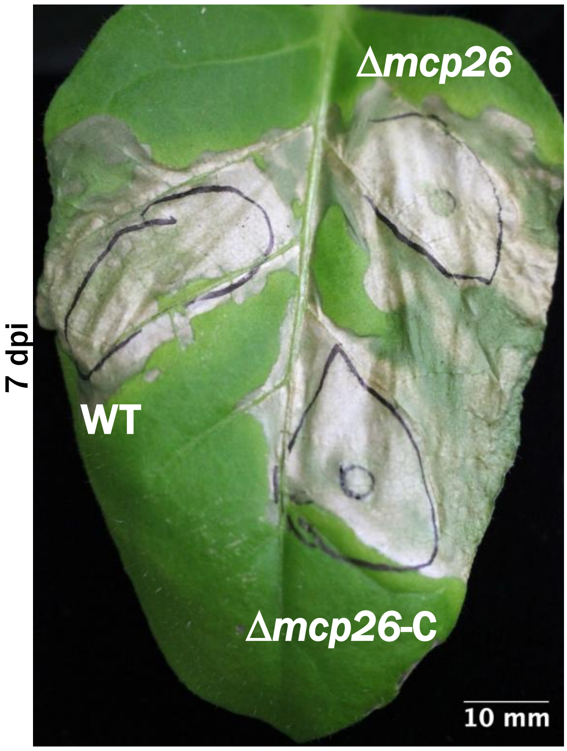

Fig. S4 Infiltration inoculation of tobacco plants

Tobacco leaves were infiltrated with bacterial suspension at a density 2×10^5 CFU/ml in 10 mM $MgSO_4$. Symptoms were observed at 7 dpi. The bar is 10 mm

```
                     *   *               *
Atu0526     110-AGEFIRLTTNLKNEKGERAAGTKLATDHPAFEKVSKGEAYF-150
PacF        137-GEDFTRITTSLKKEDGSRAMGTKLDRESPAYALVIKGETYS-177
McpF        134-GDDFVRISTSLSKQDGTRAIGTVLDRKGAAYERLIAGQSYV-174
CONSENSUS       g dF Ri TsLkkedG RA GTkLdr  pAye vikGe Y
```

Fig. S5 Multiple alignment of the ligand binding domains (LBD) of Atu0526, PacF, and McpF

LBDs of Atu0526 (AAK86341.2), PacF (WP_011095117), and McpF (Mcp26) (WP_010193774) were determined by Pfam domain PF17201 (Cache_3-Cache_2) in SMART. Multiple alignment was performed by MAFFT (ver. 7). The amino acids identical to Mcp26 are white on a black background. CONSENSUS indicates conserved amino acid sequences; capital letters denote the identical amino acids in three LBDs, and lower-case letters denote major amino acids (conserved in two LBDs). Asterisks indicate the positions of amino acid residues that bind formate (R115 in Atu0526 and R142, T145 and T158 in PacF).
